# Midregional Proadrenomedullin Can Reflect the Accumulation of Visceral Adipose Tissue—A Key to Explaining the Obesity Paradox

**DOI:** 10.3390/ijerph17113968

**Published:** 2020-06-03

**Authors:** Teruhide Koyama, Nagato Kuriyama, Ritei Uehara

**Affiliations:** Department of Epidemiology for Community Health and Medicine, Kyoto Prefectural University of Medicine, Kyoto 602-8566, Japan; nkuriyam@koto.kpu-m.ac.jp (N.K.); ruehara@koto.kpu-m.ac.jp (R.U.)

**Keywords:** visceral adipose tissue, body mass index, obesity paradox, MR-proADM

## Abstract

Background: The aim of this study was to investigate whether plasma midregional proadrenomedullin (MR-proADM) reflected body composition, such as body mass index (BMI), visceral adipose tissue (VAT), subcutaneous adipose tissue (SAT), VAT/SAT ratio, body fat mass (BFM), and skeletal muscle mass (SMM). Methods: A total of 2244 individuals (727 men and 1517 women) were included in the study. Multiple regression analysis was performed to assess the combined influence of variables: age, daily alcohol consumption, Brinkman index, sleeping time, metabolic equivalents, anamnesis for hypertension, dyslipidemia, diabetes, and body composition of MR-proADM, by using a stepwise forward selection method. Results: MR-proADM was significantly related to all anthropometric indices (BMI, VAT, SAT, VAT/SAT ratio, BFM, and SMM) in men and women. On the basis of a stepwise forward selection method, VAT (men: beta = 0.184, *p* < 0.001, women: beta = 0.203, *p* < 0.001) and BFM (beta = 0.181, *p* < 0.001) in women, were found to be significantly associated with MR-proADM. Conclusion: This study suggests that plasma MR-proADM concentration is a more reliable indicator of VAT for fat distribution, and thus, MR-proADM may help better understand the obesity paradox. Changes in circulating levels of MR-proADM could possibly reflect changes in body composition, endocrine, and metabolic milieu.

## 1. Introduction

Obesity is a complex multifactorial disease. It adversely affects nearly all physiological functions of the body and constitutes a significant public health threat [1,2,3]. Overweight and obesity rates have increased considerably during the past 35 years. Currently, more than one-third of the world’s population is classified as overweight or obese [4]. The body mass index (BMI) is a simple metric used to indicate overall body fatness. However, a phenomenon known as the “obesity paradox” occurs when a population with normal BMI possibly has a higher risk of mortality than a population with obese BMI [5,6]. In general, studies have shown that the distribution of body fat, and not the mass of body fat, is the key to explaining the obesity paradox [6]. Knowledge of the changes in body composition and fat distribution may help to understand the cause of obesity [7].

Adrenomedullin (ADM) is a vasoactive peptide identified in human pheochromocytoma [8]. ADM is secreted from various organs and tissues, and involved in several physiological functions [9,10]. ADM is also known as adipokine [11]. Previous studies also reported that the expression of ADM is augmented in the visceral adipose tissue of obese individuals than in nonobese individuals [12,13]. Plasma ADM levels were also found to be higher in obese patients than in controls, suggesting the active production and secretion of ADM from human adipose tissue. ADM was found to be positively correlated with BMI in obese participants [14], and increased ADM levels predicted a faster increase in BMI and waist circumference [15]. In sum, ADM may be a key molecule to understand body composition and fat distribution. However, to our knowledge the relationship of ADM with fat and lean tissue mass has not been studied and may require further investigation. Although reliable measurement of plasma ADM levels is difficult because of its short half-life of 22 min [16], the precursor molecule of ADM, midregional proADM (MR-proADM), has a higher stability, which allows it to be reliably measured as a surrogate biomarker for ADM [17]. Thus, to clarify whether plasma MR-proADM is representative of body composition and fat distribution, we investigated the relationship between MR-proADM and anthropometric indices, such as BMI, visceral adipose tissue (VAT), subcutaneous adipose tissue (SAT), VAT/SAT ratio, body fat mass, and skeletal muscle mass.

## 2. Materials and Methods

### 2.1. Participants

The Japan Multi-Institutional Collaborative Cohort Study was launched in 2005 to investigate the gene–environment interactions of lifestyle-related diseases [18]. This study included individuals who were enrolled in the Japan Multi-Institutional Collaborative Cohort Study’s second survey in the Kyoto area from 2013–2017 [19]. A total of 3913 participants were eligible. All subjects underwent routine health check-ups. Among the 3913 participants, 1669 were excluded because of insufficient laboratory data. After these exclusions, 2244 individuals (727 men and 1517 women) were eligible. 

The study was approved by the Institutional Ethics Committee of the Kyoto Prefectural University of Medicine (approval number: RBMR-E-36-8 at 2013) and was conducted in accordance with the principles of the Declaration of Helsinki. All participants provided written informed consent before participation.

### 2.2. Clinical and Biochemical Analysis

The following lifestyle and medical information, which was obtained through self-administered questionnaires, was evaluated: alcohol consumption (ethanol/day), Brinkman index (assessed as the number of cigarettes smoked per day × total number of years smoked), sleeping time, current medication use, and physical activity (assessed as metabolic equivalents (METs)) [20] for daily and leisure activities. The alcoholic content of each type of beverage was calculated; the alcohol consumption was determined by calculating the number of drinks per day, and subsequently, this amount was converted into the Japanese sake unit, “gou” (180 mL), which is equivalent to 23 g of ethanol. In addition, anthropometric data obtained from health check-ups were collected. Waist circumstance (WC) was measured at the umbilical level during minimal respiration in a standing position. BMI was calculated as weight divided by the square of height (kg/m^2^). 

VAT and SAT were measured using a dual bioelectrical impedance analyzer (DUALSCAN, Omron Healthcare Co. Ltd., Kyoto, Japan). The brachial–ankle pulse wave velocity (baPWV), which is used to evaluate arterial stiffness, was measured with a volume–plethysmographic apparatus (BP-203RPE II form PWV/ABI, Omron Healthcare Co. Ltd., Kyoto, Japan). We simultaneously measured baPWV on both the right and left sides, and the average of the two values for each individual supplied the population data for statistical analysis. Body composition was examined by bioelectrical impedance analysis to measure skeletal muscle mass. Model HBF-375, Omron Healthcare Co., Ltd. Kyoto, Japan, was used to measure the entire body for body fat mass (BFM) and skeletal muscle mass (SMM) [21]. Medication histories were assessed using self-administered questionnaires. Hypertension was defined as a systolic/diastolic blood pressure (SBP/DBP) ≥ 140/90 mmHg and/or current use of medication for hypertension. Dyslipidemia was defined as low-density lipoprotein cholesterol (LDL-C) ≥ 140 mg dL^−1^ and/or high-density lipoprotein cholesterol (HDL-C) < 40 mg dL^−1^ and/or current use of medication for dyslipidemia. Diabetes was defined as a glycated hemoglobin (HbA1c) level ≥ 6.5% and/or blood sugar (BS) ≥ 126 and/or current use of diabetic medication.

MR-proADM plasma concentrations were measured using an automated KRYPTOR analyzer (ThermoFisher Scientific, Germany), with a time-resolved amplified cryptate emission (TRACE) technology assay (Thermo Fisher Diagnostics K.K., Japan), according to the manufacturer’s instructions [17]. 

### 2.3. Statistical Analysis

Continuous variables are expressed as means ± standard deviation (SD) and categorical data are expressed as sums and percentages. Intergroup comparisons were performed using Welch’s t-tests for continuous variables and Fisher’s exact tests for categorical variables. Spearman’s rank correlation analysis was performed to assess the relationship between MR-proADM and other variables, including body composition. Multivariate linear regression analysis was used to test associations between MR-proADM and anthropometric indices as represented by the beta coefficient adjusted for age (continuous), daily alcohol consumption, Brinkman index, sleeping time, METs, anamnesis for hypertension, dyslipidemia, diabetes, and body composition. Multiple regression analysis was performed to assess the combined influence of variables on MR-proADM and avoid multicollinearity using a stepwise forward selection method. All statistical analyses were performed using JMP 13 software (SAS Institute Inc., Cary, NC, USA), and *p* < 0.05 was considered statistically significant.

## 3. Results

A total of 2244 participants were divided into two groups according to sex. The anthropometric measurements and participant characteristics used in this study are shown in Table 1. The mean age was 59.7 ± 10.3 years in men and 57.1 ± 9.92 years in women. The MR-proADM level differed significantly by sex, 0.467 ± 0.099 nmol/L in men versus 0.412 ± 0.082 nmol/L in women (*p* < 0.001). The SMM and METs values showed no significant difference between the populations of men and women.

Correlations between MR-proADM and body composition are given in Table 2. In men, MR-proADM was found to be significantly correlated with all indices: BMI (coefficient = 0.130), VAT (coefficient = 0.277), SAT (coefficient = 0.161), VAT/SAT ratio (coefficient = 0.225), BFM (coefficient = 0.336), and SMM (coefficient = −0.485). In women, MR-proADM was found to be significantly correlated with BMI (coefficient = 0.304), VAT (coefficient = 0.365), SAT (coefficient = 0.333), VAT/SAT ratio (coefficient = 0.062), BFM (coefficient = 0.435), and SMM (coefficient = −0.413).

In multivariable logistic regression, MR-proADM was significantly correlated with age (men: beta = 0.357, *p* < 0.001, women: beta = 0.314, *p* < 0.001), hypertension (men: beta = 0.081, *p* = 0.020, women: beta = 0.086, *p* < 0.001), daily alcohol consumption (men: beta = 0.124, *p* < 0.001, women: beta = 0.098, *p* < 0.001), and VAT (women: beta = 0.173, *p* = 0.003). These values are shown in Table 3. On the basis of a stepwise forward selection method, VAT (men: beta = 0.184, *p* < 0.001, women: beta = 0.203, *p* < 0.001) and BFM (beta = 0.181, *p* < 0.001) in women were found to be significantly associated with MR-proADM.

## 4. Discussion

The main findings of the present study can be summarized as follows. There were proven sex-based differences in the MR-proADM levels. MR-proADM was significantly related to all anthropometric indices (BMI, VAT, SAT, VAT/SAT ratio, BFM, and SMM) in men and women. Independent of age, anamnesis, and lifestyle factors, MR-proADM was significantly associated with VAT from a stepwise forward selection method. However, BMI, SAT, and VAT/SAT ratio showed no significant association with MR-proADM in both sexes. In general, studies show that fat distribution, and not fat deposit, is the key factor explaining the obesity paradox [6]. Some studies have suggested that the VAT is more strongly associated with metabolic and cardiovascular risk than SAT [22,23,24]. These results indicated that the plasma MR-proADM concentration was representative of fat distribution.

In general, men have larger amounts of VAT than women, whereas women have more subcutaneous fat areas [25,26,27]. In Table 1, the means of VAT and BFM differed significantly by sex. MR-proADM was associated with BFM, but not with SAT and VAT/SAT ratio in women. These results may indicate that increased BFM signified increased VAT in women. These sex-based differences in MR-proADM were predicted to indicate the difference in the amount of VAT accumulation.

Adipokines influence several important systemic phenomena: energy metabolism, inflammation, cancer, and infectious disease. In the process, they interact with a large number of different organ systems, vasculature, muscle, reproduction, and liver [28]; ADM is also known as an adipokine [11]. Previous reports indicate that ADM expression significantly increased obesity compared to the control group. In addition, elevated plasma ADM levels corresponded with increased body weight in animal models [29] and human studies [30]. BMI correlated with MR-proADM not only in lean participants, but also in obese patients with no additional medical problems [14]. From the prospective of weight loss, the decrease in high sensitive C-reactive protein levels during weight loss was an independent determinant of changes in plasma MR-proADM concentrations [31,32]. MR-proADM correlated with a reduction in BFM, indicating a reduced release into the circulation from adipocytes [11]. We examined the biological function of VAT among the adipocytes to explain the results of this study. Whereas many inflammatory markers were similarly associated with SAT and VAT, some inflammatory markers were more strongly associated with VAT than with SAT [33]. Some studies confirm that VAT is a major source of secreted ADM [13,14]. The increase in ADM levels found in the case of low-grade inflammation in VAT associated with obesity may be a counter-regulatory (compensatory) mechanism for inflammatory cytokines [34,35]. However, the contribution of VAT to inflammation may not be completely accounted for by clinical measures of BMI [33]. Although BMI is widely used, it does not accurately measure fat content, reflect proportions of muscle and fat, or account for sex and ethnic differences in fat content and the distribution of visceral and subcutaneous fat [36]. Thus, BMI cannot accurately reflect fat distribution. Furthermore, VAT may be a critical factor of inflammation that is not completely accounted for by BMI and waist circumference [33]. Normal weight evaluated by BMI may mask metabolically healthy or unhealthy conditions, contributing to the obesity paradox, because VAT accumulation was not determined. To our knowledge, this study is the first to report that MR-proADM is significantly associated with VAT, not BMI. Based on the pleiotropic effects of the ADM signal [9,37,38], changes in circulating levels of MR-proADM could possibly reflect changes in body composition, endocrine, and metabolic milieu.

The limitations of our study include its cross-sectional design. In a follow-up survey, we plan to assess the participants by accessing actual medical records; therefore, we expect that these additional data will lead to further detailed analysis of cardiometabolic disease. The strength of the current study is that many participants were included, and we implemented a population-based cohort design.

## 5. Conclusions

Our results suggest that plasma MR-proADM concentration is a more reliable indicator of VAT for fat distribution, and thus, MR-proADM may help better understand the obesity paradox.

## Figures and Tables

**Table 1:** Characteristics of participants according to sex.

	Men		Women		
	*n* = 727		*n* = 1517		
	Mean	SD	Mean	SD	*p*-Value
Age (years)	59.7	10.3	57.1	9.92	<0.001
BMI (kg/m^2^)	23.5	2.98	21.6	3.18	<0.001
VAT (cm^2^)	79.9	36.0	51.3	26.6	<0.001
SAT (cm^2^)	145	50.9	130	66.4	<0.001
VAT/SAT ratio	0.552	0.187	0.427	0.182	<0.001
BFM (%)	23.6	4.38	30.36	4.69	<0.001
SMM (%)	30.1	2.28	24.94	6.24	0.300
METs (hours/day)	14.8	11.0	15.4	10.4	0.248
Brinkman index	513	557	69	186	<0.001
Alcohol (g/day)	22.5	28.1	6.63	13.3	<0.001
Sleep time (hours)	6.48	1.03	6.37	1.00	0.016
MR-proADM (nmol/L)	0.467	0.099	0.412	0.082	<0.001
	*n*	%	n	%	
Hypertension	364	50.1	440	29.0	<0.001
Dyslipidemia	322	44.3	675	44.5	0.482
Diabetes	74	10.2	49	3.2	<0.001

Note: *p*-values were determined using Welch’s *t* test or Fisher’s exact tests.

**Table 2:** Correlations between midregional proadrenomedullin (MR-proADM) and anthropometric indices.

	Men		Women	
	Coefficient	*p*-Value	Coefficient	*p*-Value
BMI	0.130	<0.001	0.304	<0.001
VAT	0.277	<0.001	0.365	<0.001
SAT	0.161	<0.001	0.333	<0.001
VAT/SAT ratio	0.225	<0.001	0.062	0.015
BFM	0.336	<0.001	0.435	<0.001
SMM	−0.485	<0.001	−0.413	<0.001

**Table 3:** Comparison of the association of MR-proADM with anthropometric measures.

	Men				Women			
			Stepwise				Stepwise	
	Beta	*p*-Value	Beta	*p*-Value	Beta	*p*-Value	Beta	*p*-Value
Age	0.357	<0.001	0.415	<0.001	0.314	<0.001	0.278	<0.001
Hypertension	0.081	0.020	0.086	<0.001	0.086	<0.001		
Dyslipidemia	−0.008	0.804			−0.021	0.387		
Diabetes	0.019	0.566			0.015	0.490		
Sleep time	−0.019	0.564			0.020	0.375		
Alcohol	0.124	<0.001	0.124	<0.001	0.098	<0.001	0.110	<0.001
Brinkman index	0.028	0.419			0.010	0.676		
METs	−0.045	0.160			−0.062	0.005		
BMI	−0.058	0.349			0.048	0.340		
BFM	0.080	0.381			−0.039	0.608	0.181	<0.001
SMM	−0.052	0.533			−0.061	0.262		
VAT	0.130	0.326	0.184	<0.001	0.173	0.003	0.203	<0.001
SAT	0.009	0.936			0.138	0.016		
VAT/SAT ratio	0.011	0.902			−0.027	0.551		

Note: beta standardized regression coefficients.
